# Home oxygen therapy for Thai preterm infants with bronchopulmonary dysplasia. What are the predictive factors for successful weaning: a 20-year review

**DOI:** 10.1186/s12887-024-05354-1

**Published:** 2025-01-11

**Authors:** Vipada Grajangdara, Anchalee Limrungsikul, Allan L. Coates, Harutai Kamalaporn

**Affiliations:** 1 Department of Pediatrics, Faculty of Medicine, Ramathibodi Hospital, Mahidol University, Bangkok, Thailand; 2 Division of Neonatology, Department of Pediatrics, Faculty of Medicine, Ramathibodi Hospital, Mahidol University, Bangkok, Thailand; 3 The Research Institute, Hospital for Sick Children, University of Toronto, Toronto, Canada; 4 Division of Pulmonology, Department of Pediatrics, Faculty of Medicine, Ramathibodi Hospital, Mahidol University, Bangkok, Thailand

**Keywords:** Bronchopulmonary dysplasia, Home oxygen, Preterm infants, Oxygen withdrawal, Resource poor country

## Abstract

**Background:**

Consequences of lung injury and inflammation in preterm infants with bronchopulmonary dysplasia (BPD) contribute to prolonged oxygen requirements. Home oxygen therapy (HOT) is an alternative way of respiratory support in infant with BPD. However, there is no consensus on weaning guidelines. Our objective is to identify the median age of HOT discontinuation and the factors that might predict the duration of HOT in a resource poor country.

**Methods:**

All preterm (≤ 36 weeks’ gestation) infants diagnosed with BPD who required HOT after discharged from Ramathibodi Hospital during January 2000 – December 2019 comprised this retrospective study. Timing of HOT withdrawal was identified. Demographic data, severity of BPD, maternal condition, respiratory support, comorbidities, complications, and growth were recorded and analyzed as factors associated of home oxygen withdrawal.

**Results:**

Of 8581 preterm infants born during the 20-year period, 563 (6.6%) had BPD. Among 40 infants treated with HOT, 18 (45%) were successfully weaned from oxygen within 12 months. The median corrected age (CA) of oxygen withdrawal was 13.8 months (8.5, 22.1). Longer duration of total respiratory support, longer length of hospital stay and poor growth determined by weight, length and head circumference were associated with longer duration of HOT. Greater weight gain was associated with a shorter duration of HOT at 12 months CA (adjusted OR, 1.97; 95% CI, 1.13–3.23; *p* = 0.015).

**Conclusions:**

The median corrected age of oxygen withdrawal in Thai BPD infants was 13.8 months. Severe BPD and poor linear growth were associated with prolonged HOT.

## Summary at a glance

Home oxygen therapy (HOT) for the treatment of bronchopulmonary dysplasia in resource poor countries is costly for families. This 20-year review which includes both the pre surfactant era and the post gives some predictive guidelines into the timing for discontinuing HOT, hence helping families with economic planning.

## Introduction

Bronchopulmonary dysplasia (BPD) is a common complication in premature infants requiring mechanical ventilation. While advances in neonatal care increases survival at earlier gestational ages (GA), chronic neonatal respiratory disease remains a challenge. BPD is now characterized by impairment of alveolarization resulted from immaturity more than lung injury from mechanical ventilator [1–6]. The 2001 definition of BPD as a requirement for more than 28 days of supplemental oxygen (O_2_) and severity defined by need for O_2_ or positive pressure support at 36 weeks GA if born at < 32 weeks GA, 56 days chronological age, or at the time of discharge if born at ≥ 32 weeks GA, is simple but may be outdated [2, 4–10].

The main endpoint of O_2_ therapy in BPD patients is to maintain target O_2_ saturation of 90–95% and to prevent pulmonary hypertension (PH) [11–14]. If weaning to room air cannot happen before discharge, an alternative is to give O_2_ at home which improves the quality of life, prevents nosocomial infection and saves costs [14, 15]. There are some available guidelines or statements in which most recommendations focus on initiation or indication of home oxygen in BPD [16–22], however, only a few mention about the weaning [23–25]. Although home O_2_ therapy (HOT) is worldwide, there is no consensus on weaning guidelines either under the supervision of physicians or unsupervised weaning by parents [15, 16, 18, 23, 24]. European Respiratory Society (ERS) guidelines on management of children with BPD has suggested the “O_2_ reduction test” assessing O_2_ saturation during weaning to room air. The utility of home O_2_ saturation monitoring is unclear [18]. In United States, weaning is recommended to be under the supervision of a pediatric pulmonologist using pulse- oximetry while awake with subsequent weaning during sleep assessed through overnight polysomnography or home pulse-oximetry. However, in a study by Yeh et al. [23] home O_2_ withdrawal without physicians’ supervision was observed in 32% of children with BPD. Studies have shown a median age, from 2 to 12.5 months, when HOT was discontinued [13, 22, 24–28]. Factors associated with earlier HOT discontinuation were shorter duration of neonatal intensive care unit (NICU) stay [26] and lower flow requirements [23]. There are various O_2_ generator devices and different assessment modes of O_2_ weaning [16] but no practical guidelines for HOT weaning in a resource- poor country.

At Ramathibodi Hospital, a tertiary care center in Thailand, a HOT program for children with BPD has been active for more than 20 years. Our multidisplinary very-low-birthweight (VLBW) clinic includes neonatologists, pediatric pulmonologists, gastroenterologists, nutritionists and nurse coordinators. The pediatric pulmonologists consider O_2_ weaning based on physical examination, O_2_ saturation in room air, parental record of pulse oximetry monitoring at home > 92% with various activities in patients without PH, > 95% in patients with PH, and adequate weight gain. While many guidelines stress continuous home O_2_ monitoring, especially while asleep [20], the equipment to do this was beyond the financial resources of our families. Some physicians may perform O_2_ stress test or O_2_ reduction test. Due to resource limitations overnight pulse oximetry or polysomnography are reserved for the patients with complications such as PH, bronchomalacia, subglottic stenosis or patients with home ventilators. The O_2_ delivery systems included O_2_ tanks and O_2_ concentrators delivered by nasal prongs, available since 2010. While the O_2_ concentrator was convenient and less expensive, it was impossible to titrate O_2_ flows accurately below 1 L/min. Thai system of national health coverage does not include HOT and has less resources for evaluating readiness for weaning. Low flow O_2_ devices and polysomnography are not available in most of the hospitals.

With the economic burden, the most frequently asked question from parents is when they could expect the HOT withdrawal. Thus, to provide prognostic information for parents, the aim of this 20-year retrospective study was to identify the median age of HOT discontinuation and the factors that might predict prolonged HOT. Comparisons of the outcomes such as mortality and readmission to the resource- rich countries were the secondary objectives.

## Materials and methods

### Study population

This retrospective study was performed in the division of pediatric pulmonology, Faculty of Medicine, Ramathibodi Hospital, Bangkok, Thailand from January 2000 - December 2019. Electronic medical records of infants with BPD who were discharged with HOT were reviewed. The 2001 definition of BPD [4] was used since it was the accepted definition at start of the study. All preterm infants born before 36 GA, diagnosed with BPD, and followed up in the VLBW and chest clinic were recruited. We excluded patients who required HOT due to other causes such as congenital cyanotic heart diseases, congenital airway anomalies, or congenital diaphragmatic hernia. The need for consent to participate was deemed unnecessary for retrospective chart review according to national regulations. This study adheres to the ethical standards, with all necessary approvals obtained from the Human Research Ethics Committee, Faculty of Medicine Ramathibodi Hospital, Mahidol University (COA. MURA2020/901).

### Outcomes

The primary outcome was the median corrected age (CA) when home O_2_ use was discontinued. O_2_ withdrawal was defined as either under the supervision of a physician or the unsupervised discontinuation by parents.

Demographic and clinical data including GA, prenatal history, severity of BPD, history of surfactant administration, duration of mechanical ventilation and O_2_ supplementation, procedures and medications, growth assessment, comorbidities, and complications were recorded. Small for gestational age (SGA) was defined as birth weight < 10th percentile based on Fenton 2013 preterm growth chart [29]. The rate of weight gain at O_2_ withdrawal was calculated from the subtraction of weight at the time of discharge and the time of O_2_ withdrawal divided by the duration of HOT. The velocity of growth parameters such as length and head circumference were calculated in the same manner. The method of weaning and evaluation of O_2_ cessation were included.

### Statistical analyses

Baseline characteristics were tested for normality by Kolmogorov-Smirnov or Shapiro-Wilk test and presented as means with standard deviation or medians with interquartile range. Mann-Whitney *U* test and *t*-test were used to compare continuous variables with non-normally distributed and normally distributed, respectively, while chi-square was used to compare categorical variables. Adjusted odd ratios were assessed by logistic regression analysis to identify correlation between possible prognostic factors and the corrected age of O_2_ withdrawal at 12 months. Statistical significance was considered when P-values < 0.05.

The 20-year delivery period was divided into 2 periods (2000 to 2009, the pre-surfactant era and 2010 to 2019, the post-surfactant era) with regard to differences of home O_2_ use. The associations were analyzed based on univariate and multivariate logistic regression analysis.

## Results

**Fig. 1:**
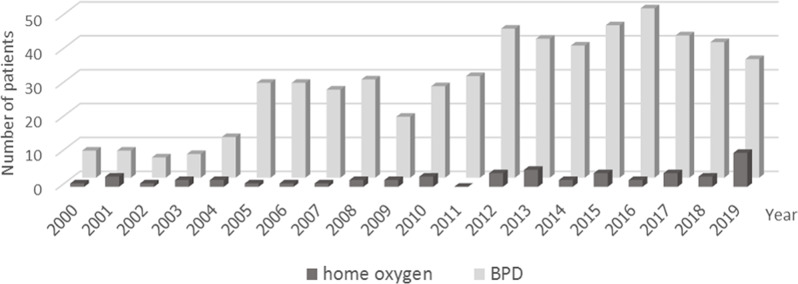
The number of infants with BPD and those who were sent home with oxygen supplementation during the year 2000–2019

During January 2000 – December 2019, 8581 preterm infants were born at Ramathibodi Hospital. The prevalence of BPD was 563 of 8581 (6.6%) of the preterm infants (Fig. 1).

Fifty-three of 563 (9.4%) infants with BPD were discharged home on HOT. Thirteen patients were excluded; 2 patients died before the O_2_ cessation (the first patient died at 4 months CA from severe pneumonia, another died from severe pulmonary hemorrhage and PH at 7 months CA), 2 patients had congenital diaphragmatic hernia, 1 had Pierre- Robin syndrome, and 8 patients were lost to follow- up. The remaining 40 BPD patients who received HOT were recruited. The baseline characteristics of infants with BPD and those who were sent home on HOT during the year 2000–2019 is shown in Table 1.

**Table 1:** Baseline characteristics

Variables	Home oxygen (*n* = 40)
Male sex (%) Gestational age (weeks) ^a^ Birth weight (g) ^b^ Small for gestational age (%) Low APGAR (%) Cesarean section (%)	15 (38) 27.9 ± 2.5 845 (722.5, 1040) 13 (32.5) 6 (15) 28 (70)
**Maternal condition**	
chorioamnionitis (%) Maternal diabetes (%) Maternal pregnancy-induced hypertension (%) Antenatal steroids (%) Premature rupture of membrane (%)	4 (10 3 (7.5) 15 (37.5) 26 (65) 10 (25)
**Respiratory support**	
Surfactant (%) Oxygen required at delivery (%) Invasive ventilation (days) ^b^ Noninvasive ventilation (days) ^a^ Total respiratory support (days) ^b^ High-frequency oscillatory ventilation (%) High-frequency oscillatory ventilation (days) ^b^	28 (70) 36 (94.7) 29 (14, 38) 68 ± 39 146 (126, 178) 29 (72.5) 12 (6, 22) (*n* = 29)
**BPD and management**	
Severe BPD (%) Inhaled corticosteroids (%) Bronchodilator (%) Diuretics (%) Surgical PDA ligation Length of hospital stay (days) ^a^ CA at discharge (weeks) ^b^ CA at O_2_ withdrawal (months) ^c^ Duration of home O_2_ therapy (months) ^c^	35 (87.5) 26 (65) 11 (27.5) 36 (90) 6 (15) 159 ± 48 8.6 (5.3, 12.6) 13.8 (8.5, 22.1) 11.3 (5.6, 17.5)

*Abbreviations* CA, corrected age; O_2_, oxygen; PDA, patent ductus arteriosus; SGA, small for gestational age defines as birth weight < 10th percentile based on Fenton 2013 preterm growth chart.

^a^ Continuous variables were expressed as mean ± standard deviation, ^b^ Continuous variables were expressed as median (interquartile range), ^c^ Continuous variables were expressed as median (interquartile range) excluded infants who continued oxygen use (*n* = 37)

The subjects had a mean GA of 27.9 ± 2.5 weeks and a median birth weight of 845 g (722.5, 1040). Thirty-five (87.5%) had severe BPD. The total length of hospital stay was 159 ± 48 days. Twelve subjects (30%) had echocardiogram confirmed PH.

**Fig. 2:**
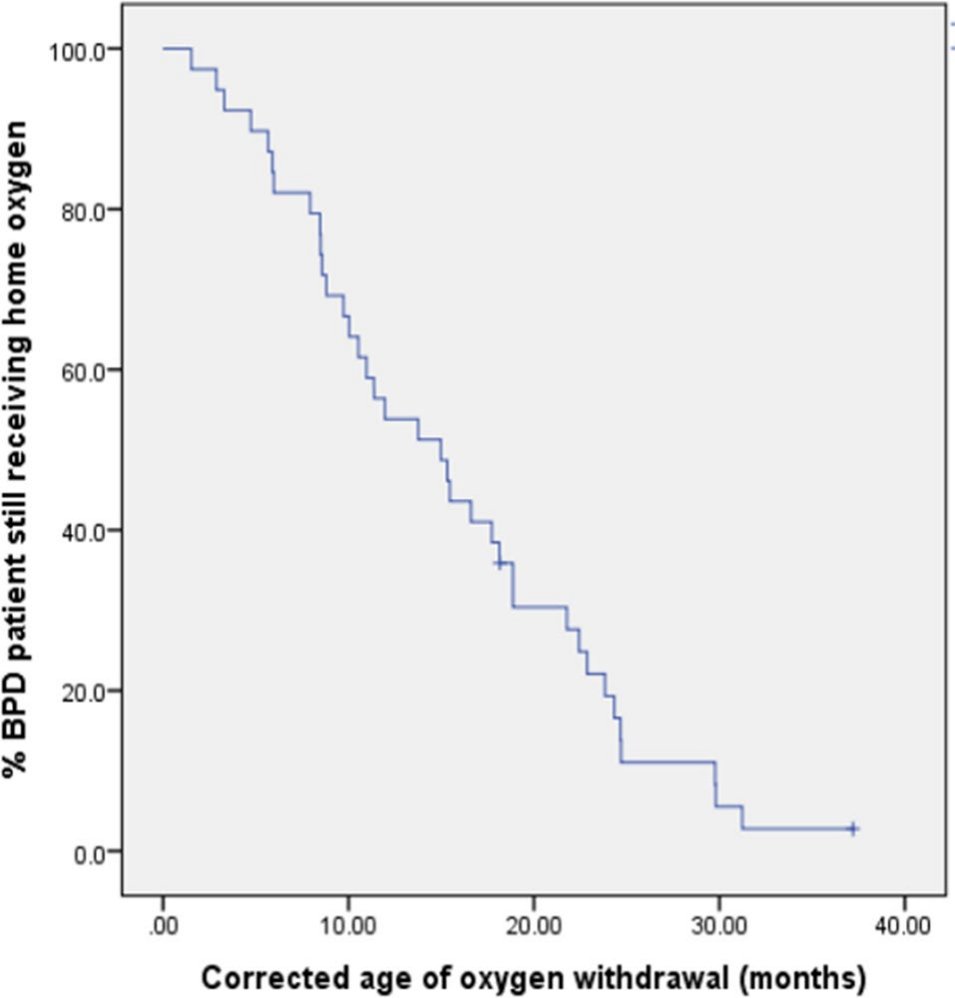
Kaplan-Meier Plot demonstrating the age of oxygen withdrawal in 40 BPD infants

Of the 40 infants, 37 (92.5%) discontinued HOT during the study period and 18 of 40 infants (45%) successfully weaned off O_2_ by 12 months (Table 2; Fig. 2).

**Table 2:** Comparisons of factors in BPD infants with successful HOT discontinuation at 12 months CA cutoff

Factors	O_2_ withdrawal ≤ CA 12 mo (*n* = 18)	O_2_ withdrawal > CA 12 mo (*n* = 22)	*P*-value
Gestational age (weeks) ^b^ Birth weight (g) ^b^ Low APGAR (%) Maternal diabetes (%) Maternal pregnancy-induced hypertension (%) Antenatal steroids (%) Invasive ventilation (days) ^a^ Noninvasive ventilation (days) ^a^ HFOV (%) HFOV (days) ^b^ Maximum MAP of HFOV ^a^ Severe BPD (%) Inhaled corticosteroids (%) Diuretics (%) Bronchodilators (%) Length of hospital stay (days) ^b^ CA at discharge (weeks) ^b^ Weight at discharge (g) ^a^ Weight at O_2_ withdrawal (g) ^a^ Weight gain at O_2_ withdrawal (g/day) ^a^	28.4 (26.7, 30.7) 965 (710, 1165) 4 (22.2) 2 (11.1) 8 (44.4) 15 (83.3) 26 ± 16 46 ± 27 11 (61.1) 12 (4, 16) 14 ± 2.7 13 (72.2) 10 (55.6) 16 (88.9) 5 (27.8) 126 (111, 170) 5.9 (4, 11.4) 4291 ± 897 7083 ± 1818 17 ± 7.3	27.1 (25.9, 28.6) 825 (713, 945) 2 (9.1) 1 (4.5) 7 (31.8) 19 (86.4) 34 ± 25 85 ± 40 18 (81.8) 13 (6, 23) 15.4 ± 3.1 22 (100) 16 (72.7) 20 (90.9) 6 (27.3) 161 (134, 211) 10.1 (7.6, 17) 5178 ± 960 10,072 ± 1330 8.8 ± 2.6	0.138 0.192 0.381 0.579 0.412 1.000 0.242 0.001 0.173 0.589 0.226 0.013 0.257 1.000 1.000 0.008 0.019 0.005 < 0.001 < 0.001

*Abbreviation* HFOV, High-frequency oscillatory ventilation; MAP, mean airway pressure; CA, corrected age; O_2_, oxygen; DOL, day of life; HHHFNC, Heated humidified high flow nasal cannula; ICS, inhaled, ^a^Continuous variables were expressed as mean ± standard deviation; ^b^ Continuous variables were expressed as median (interquartile range)

Table 2 lists the possible factors associated with O_2_ withdrawal at 12 months CA. Shorter length of hospital stay (126 vs. 161 days, *p* = 0.008), higher weight gain at O_2_ withdrawal (17 ± 7.3 vs. 8.8 ± 2.6 g/day, *p* < 0.001) and lower proportion of severe BPD (72.2% vs. 100%, *p* = 0.013) were associated with successful HOT weaning at or before 12 months CA. Multivariate logistic regression analysis revealed that only the higher rate of weight gain remained a significant factor for O_2_ withdrawal at 12 months CA (adjusted OR, 1.91; 95% CI, 1.13–3.23; *p* = 0.015). The comparisons of growth velocity determined by weight, length, and head circumference between the two groups were as shown in Table 3

**Table 3:** Comparisons of growth velocity and duration of successful HOT withdrawal

Growth velocity	O_2_ withdrawal	O_2_ withdrawal	*p*- value
	≤ CA 12 mo	> CA 12 mo	
**At the starting point of weaning**			
Weight gain (g/day)	20 (14.7, 28.4)	7.1 (3, 71)	< 0.001
Length (cm/day)	0.08 (0.07, 0.13)	0.04 (0.03, 0.09)	0.027
Head circumference (cm/day)	0.03 (0.02, 0.06)	0.01 (0.01, 0.02)	0.047
**At the point of successful weaning**			
Weight gain (g/day)	13.2 (6.7, 19.4)	5.1 (3.9, 11.1)	0.013
Length (cm/day)	0.06 (0.05, 0.08)	0.02 (0.01, 0.03)	0.003
Head circumference(cm/day)	0.02 (0.02, 0.05)	0.01 (0, 0.01)	< 0.001

The median CA of O_2_ withdrawal was 13.8 months (8.5, 22.1) and the median was 11.3 months (5.6, 17.5). There were six patients with prolonged HOT beyond the corrected age of 2 years. All of them had severe BPD and had been treated with high-frequency oscillatory ventilation (HFOV). The characteristics of these patients are shown in Table 4.

**Table 4:** Characteristics of the BPD patients with prolonged home oxygen therapy beyond the corrected age of 24 month

Subject No.	Year of birth	GA (wk)	BW (g)	PPHN	LOS (d)	Readmission (*n*)	Age at O_2_ withdrawal (mo)	Remarks*
1	2009	34	720	No	232	0	24.3	bronchomalacia, atelectasis, GERD
2^a^	2013	25	845	Yes	225	2 (PICU)	29.8	pulmonary hypertension, subglottic stenosis, GERD
3^a^	2014	27	770	No	146	3	24.7	BBA
4	2017	26	800	No	184	1	31.2	Triplets, BHR
5^a^	2018	25	675	Yes	135	6 (1 PICU)	37.2	GERD, aspiration pneumonia
6	2018	29	930	Yes	133	1 (PICU)	29.8	bronchomalacia, ARDS, subglottic stenosis, GERD

*Abbreviations* GA, gestational age; BW, birth weight; PPHN, persistent pulmonary hypertension of the newborn; LOS, length of hospital stay; O_2_, oxygen; CA, corrected age; ARDS, acute respiratory distress syndrome; GERD, gastroesophageal reflux disease; BHR, bronchial hyperresponsiveness; BBA, birth before arrival

*All patients had severe BPD

^a^ Patients has never received antenatal steroids

The overall survival rate of those born weighing < 2500 g in the pre-surfactant era was 94.0% vs. 96.4% in the post-surfactant era (*p* < 0.01) while the incidence of BPD in these group was 4.2 and 8.4% respectively (*p* < 0.005). However, the pre surfactant babies were 2 weeks more mature than the post and weighed significantly more Table 5. The modality of respiratory support including the use of inhaled corticosteroids (ICS) differed due to changes in care over time.

**Table 5:** Comparison of infants required home oxygen therapy who were born during 2000–2009 and 2010–2019

Variables	Year 2000–2009 (*n* = 9)	Year 2010–2019 (*n* = 31)	*P*-value
Gestational age (weeks) ^a^ Birth weight (g) ^b^ SGA (%) Low APGAR (%) Maternal chorioamnionitis (%) Maternal diabetes (%) Maternal pregnancy-induced hypertension (%) Antenatal steroids (%) Surfactant administration (%) Invasive ventilation (days) ^b^ Noninvasive ventilation (days) ^a^ Total respiratory support (days) ^b^ HHHFNC (%) HFOV (%) HFOV (days) ^a^ Maximum MAP of HFOV ^a^ Severe BPD (%) Inhaled corticosteroids (%) Diuretics (%) Bronchodilators (%) Length of hospital stay (days) ^b^ Weight at discharge (g) ^a^ CA at O_2_ withdrawal (months) ^a^ Duration of home O_2_ (months)^a^ Weight at O_2_ withdrawal (g) ^a^ Weight gain at O_2_ withdrawal (g/day) ^b^ Overnight SpO_2_ monitoring (%) Oxygen concentrator use (%) Readmission due to respiratory illnesses (%)	29.7 ± 2.8 1100 (825, 1355) 3 (33.3) 2 (22.2) 0 (0) 2 (22.2) 3 (33.3) 7 (77.8) 1 (11.1) 28 (7, 31) 37 ± 25 145 (119, 174) 0 (0) 3 (33.3) 5 ± 2 10.3 ± 3.5 8 (88.9) 3 (33.3) 8 (88.9) 6 (66.7) 150 (118, 180) 4756 ± 613 9.9 ± 7 7.3 ± 6 7123 ± 1739 14.7 (5.8, 21.4) 4 (57.1) 0 (0) 4 (44.4)	27.4 ± 2.3 820 (690, 990) 10 (32.3) 4 (12.9) 4 (13.3) 1(3.2) 12 (38.7) 27 (87.1) 27 (87.1) 29 (16, 39) 77 ± 38 146 (127, 181) 29 (93.5) 26 (83.9) 14 ± 9 15.5 ± 2.5 27 (87.1) 23 (74.2) 28 (90.3) 5 (16.1) 145 (126, 179) 4786 ± 1123 16.8 ± 8.6 14.5 ± 8.2 9033 ± 2119 10.3 (7.9, 15.6) 15 (51.7) 30 (96.8) 20 (64.5)	0.016 0.041 1.000 0.602 0.560 0.121 1.000 0.602 < 0.001 0.438 0.006 0.503 < 0.001 0.007 < 0.001 0.003 1.000 0.044 1.000 0.007 0.871 0.939 0.033 0.019 0.047 0.484 0.074 < 0.001 0.441

*Abbreviations* HFOV, High-frequency oscillatory ventilation; MAP, mean airway pressure; CA, corrected age; O_2_, oxygen; DOL, day of life; HHHFNC, heated humidified high flow nasal cannula; ICS, inhaled corticosteroid; SpO_2_, oxygen saturation ^a ^Continuous variables were expressed as mean ± standard deviation, ^b^ Continuous variables were expressed as median (interquartile range)

Preterm infants delivered during 2010–2019 had longer duration of HOT compared with those born during 2000–2009 (14.5 vs. 7.3 months, *p* = 0.019) and almost all used an O_2_ concentrator (97% vs. 0 (*p* < 0.001)) (Table 5). These differences did not extend to the use of overnight oximetry monitoring (57.1% vs. 51.7%, *p* = 0.074). The hospital admission due to respiratory tract infection and BPD complication, such as PH leading to heart failure and fluid intolerance, were not significantly different.

After discharge home, there were 51 respiratory readmissions including pneumonia, atelectasis, ARDS in 24 patients. PH was diagnosed in 3 patients.

## Discussion

The median CA of children with BPD discharged from supplemental O_2_ was 13.8 months, longer than previous studies where CA at O_2_ discontinuation was 2 to 12.5 months which was likely due to different patient characteristics, mode of O_2_ support and weaning protocols [13, 22–25, 27, 28, 30, 31]. The most recent study in 2021 reported that the median CA at O_2_ cessation of 149 babies was 6.8 months with 87.2% of infants weaned by 12 months CA [26] compared to 45% (18 of 40) in our study. In contrast with Wong et al. [26] study in 2021, we had a higher proportion of SGA (32.5% vs. 8.7%), need longer respiratory support (146 vs. 73 days) and higher proportion of using HFOV (72.5% vs. 53%).

The analysis was divided into 2 time periods because the surfactant and non-invasive ventilation, especially the nasal CPAP and the heated humidified high flow nasal cannula, were widely used in NICU after 2009. Comparing time periods, (2000–2009 vs. 2010–2019) (Table 4), infants born during 2010–2019 required longer HOT (9.9 vs. 16.8 months). Despite the attempt to reduce barotrauma, use of surfactant and ICS, the proportion of severe BPD remained unchanged, presumably because infants born in the latter period had earlier GA and lower birth weight. There was a slight but significant increase in survival during the later period which may also have led to a higher incidence of BPD. For our NICU, a “rescue mode” of using high-frequency oscillatory ventilation (HFOV) was activated if the positive inspiratory pressure (PIP) > 26 cmH_2_O. Besides the use of surfactant, in 2010 INSURE technique (intubation-surfactant then extubation to CPAP) in infants with RDS who required CPAP > 7 cmH_2_O, FiO_2_ > 0.3 was used. We also used DART protocol (Dexamethasone: A Randomized Trial) prior to extubation in preterm infants who were intubated > 7 days. Ventilator-derived CPAP with constant flow and NIPPV had been used until 2010 whereas 2010–2012 was a transitional period. After 2012, variable flow CPAP has been using for almost all patients either post-extubation or primary mode for NICU respiratory support. None of prednisolone protocols was used in our institute. For the severe patients, the prevalence is lower in the most recent years likely due to using more non-invasive ventilation in NICU. Hence the prevalence of BPD is lower.

From previous studies [13, 19, 22–28, 30, 31], as many as 36.7–65.2% of preterm infants with BPD were treated with HOT whereas only 9.4% (53 of 563) in our center (Fig. 1). The explanation of such a small population in our study was that the expense of HOT is not included in the national health coverage and some parents whose children were eligible for HOT were not be able to afford it. If the parent could not afford home O_2_ the team made great efforts to find some alternative funding. As one of the poor-resource countries, usually the duration of oxygen weaning trial in the hospital in infants with BPD is 4–6 weeks before committing the patient to HOT. Due to economic reasons, some patients needed to stay in the hospital until fully weaned off oxygen. Hence, only the more severe BPD patients who could afford the costs were included in our study which may lead to the prolonged use of HOT. A multicenter study in China reported the rate of HOT use 26.8% with the negative correlation between the use of HOT and the provincial economic status. Chinese parents with lower economic level preferred early discharge with HOT due to lack of health insurance coverage or inadequate financial support for hospitalization expense [28].

The most common equipment of home O_2_ used in Thailand was O_2_ concentrator, available in the last half of the study period. It delivered O_2_ flows ranging from 0.5 to 10 L/min, typically 1–2 L/min. The British Thoracic Society guidelines propose O_2_ concentrators for long term HOT and compressed O_2_ with a low flow regulator at a rate of 0.1–1 L/min for the short anticipated duration of HOT [16]. The median discharge O_2_ flow in Wong et al. study was only 0.25 L/min.^26^ Most of participants (89%) in Yeh et al. study required less than 0.5 L/min of O_2_ supplement at discharge [23]. In US, the recommended home O_2_ flow starts from 1/8 L/min or 0.1 L/min depends on the available devices [24, 32]. In Thailand, we sent most of the babies home with at least 1 L/min of home O_2_ because the low flow O_2_ was not widely available.

Our study and Wong et al. [26] used overnight pulse oximetry monitoring as the assessment method of weaning while Yeh et al. [23] and Saletti et al. [25] used polysomnography. Only one study in 2004 reported the rapid weaning at 2 months CA by abrupt cessation of oxygen at the out-patient department when the level of oxygen saturation was more than 95% for 15 min [25]. The duration of HOT more than 12 months CA was associated with more severe BPD, longer duration of respiratory support, longer hospital stay and infants with poor weight gain (Table 2) as has been seen previously [26]. Hypercarbia on capillary blood gas has been associated with prolonged O_2_ need [33] but was not performed in our study although we monitored end tidal CO_2_ (EtCO_2_). EtCO_2_ rarely contributed to decisions that were not apparent from the SpO_2_. While higher O_2_ flow at discharge was a predictor of later home O_2_ weaning [23, 26], the O_2_ flow at discharge in our study did not predict the age of weaning.

The room air challenge (RAC) is optionally performed at 36-weeks PMA [34] or at 6 months post discharge [20] which may help provide anticipatory guidance for HOT. Failing RAC may predict longer oxygen requirement.

At our institute, almost all of the patients with HOT use home pulse oximeters with intermittent monitoring. The parents were trained to assess increased work of breathing and/or cyanosis with various activities such as crying/ feeding/defecating or sleeping and to record it sporadically. The pediatric pulmonologist saw the patient every 1–3 months to guide the weaning according to the record of home oxygen and heart rate and physical exams including growth assessment (weight, height, and head circumference). Chart reviews and meeting to have a consensus on weaning of our BPD patients before seeing them in our Chest clinic were routinely performed on a weekly basis. Diuretics weaning occurred prior to oxygen weaning, similar to a study by Palm K et al. when the questionnaires were sent to pediatric pulmonology program in US and 50% of respondents reported that they wean off diuretics first, then oxygen [35].Usually, the weaning readiness was considered when BPD infants have positive trends in growth and developmental milestones, a period of stable health condition without re-hospitalization, stable vital signs and when the record of oxygen saturation at home remained in the acceptable range (95% and above) while on oxygen 0.5–1 L/min. If the patient passed the O_2_ reduction test in the clinic the HOT period was gradually decreased from 24 h to nocturnal plus nap until successfully wean off to night time only, similar to the American Thoracic Society (ATS) clinical practice guidelines [20]. Then the SpO_2_ and EtCO_2_ were monitored in hospital before discontinuing HOT.

Supplemental O_2_ improves growth in infants with BPD [6, 17, 20, 27]. Intermittent hypoxemia has been shown to be associated with poor growth [14, 36, 37]. The proposed home O_2_ weaning protocol suggests a slower wean for if growth is stagnant [17, 19, 27, 37–39]. Our study would support these finding with the association between early O_2_ withdrawal and greater rate of weight gain. We examined the linear growth/length as well as the weight gain as we realized that linear growth is more predictive in lung growth. We discovered the association between HOT weaning outcomes and the velocity of growth (weight, length, head circumference) (Table 3).

Most of BPD children should outgrow the need for supplemental O_2_ by 2 years of life [14, 26]. Weaning duration may vary due to multiple factors such as prematurity, severity of BPD, growth, airway hyperreactivity, pulmonary hypertension, parental preference and social circumstances [30, 32, 40]. We had six patients who required prolonged HOT (Table 4). All of them presented with severe BPD. Five patients were born during 2013–2018 and had many complications such as bronchomalacia, gastroesophageal reflux disease, PH and some had subglottic stenosis. The repeated infections and inflammations led to frequent readmission and subsequent weaning difficulty.

Previous studies have shown that BPD infants who required HOT were more likely to be re-hospitalized for respiratory illness (38% vs. 28%; adjusted relative risk 1.33; 95% CI 1.16 to 1.53) [26]. Sun L et al. reported 48 of 62 (77%) BPD children had *≥* 2 readmissions within 18–24 months [10] which is comparable to our study which revealed 24 of 40 (60%) BPD patients with HOT resulted in respiratory re-hospitalization. While a study from Taiwan demonstrated higher prevalence of ED visits and re-hospitalization in BPD patients with HOT than ones without HOT within one year of corrected age [31]. A study in 2020 reported 34% of readmission among BPD infants, however, HOT is not a factor associated with readmission [37]. BPD children needing HOT were more likely to have hyperreactive airway (81% vs. 54%) compared to those who were discharged home without HOT [30].

Interestingly, the prevalence of PH in our population was lower than previous studies [30]. This may be explained by the higher flow of O_2_ provided and/or the sick infants with PH in our setting were not discharged. A pediatric cardiologist was consulted for all infants with BPD requiring HOT to diagnose a BPD-associated pulmonary hypertension (BPD-PH) at 36 weeks post menstrual age. However, the decision to perform an ECHO depended on the cardiologist’s assessment.

This study’s strength is a long duration of study period and comparisons between the pre-surfactant and aggressive ventilation era vs. the post-surfactant and non-invasive ventilation era. Limitations are due to the small sample size, a retrospective study design, missing data on radiography and socioeconomic factors and potential selection bias due to inconsistency from lack of strict guidelines such that not every infant underwent ECHO screening for pulmonary hypertension as well as weaning variations among the pediatric pulmonologists.

Using the NIH 2001 definition, the prevalence of BPD was higher than using NICHD 2018 definition. Mild BPD using 2001 criteria may not be diagnosed compared to 2018 criteria [9, 10]. However the majority (87.5%) of our subjects had severe BPD which is not significantly different between the 2 definitions.

## Conclusion

This 20-year review discovered that the median corrected age of HOT withdrawal in Thai infants with BPD was 13.8 months. Prolonged O_2_ requirement was associated with severe BPD, longer length of hospitalization and poor linear growth. Resource poor countries can have a successful HOT weaning program despite the lack of availability of sophisticated monitoring systems such as polysomnography.

## Data Availability

No datasets were generated or analysed during the current study.

## Abbreviations

BPD: Bronchopulmonary dysplasia
HOT: Home oxygen therapy
CA: Corrected age
GA: Gestational age
SGA: Small for gestational age
NICU: Neonatal intensive care unit
VLBW: Very-low-birth weight
PH: Pulmonary hypertension
RAC: Room air challenge
